# GIS Analysis of Land Cover Changes on the Territory of the Prokuplje Municipality

**DOI:** 10.1155/2014/805072

**Published:** 2014-08-03

**Authors:** Aleksandar Valjarević, Dragica Živković, Dragana Valjarević, Vladica Stevanović, Jelena Golijanin

**Affiliations:** ^1^Department of Geography, Faculty of Natural Science and Mathematics, University of Kosovska Mitrovica, Lole Ribara 29, 38220 Kosovska Mitrovica, Serbia; ^2^Faculty of Geography, University of Belgrade, Studentski trg 3/II, 11000 Belgrade, Serbia; ^3^Department of mathematics, Faculty of Natural Sciences and Mathematics, University of Kosovska Mitrovica, Lole Ribara 29, 38220 Kosovska Mitrovica, Serbia; ^4^Department of Geography, Faculty of Philosophy, University of East Sarajevo, 71126 Istočno Sarajevo, Bosnia And Herzegovina

## Abstract

The monitoring of the territory of Prokuplje Municipality was done based on 1 : 25,000 topographic maps in three different time periods (1969, 1974, and 1984) and land cover map in 2012. Analogous topographic maps done in 1969, 1974, and 1984 were used, while in 2012 the land cover map obtained by using CORINE-like approach was used. Topographic maps are developed by aerial campaign, and today they are replaced by satellite images. Topographic maps were scanned, and raster form was transformed to vector data with Geo Media Professional 6.1 and Global Mapper software. The monitoring in the period of 1969–2012, on the area of 758300000 m^2^, was performed, where some parameters were analyzed. In particular, the changes of natural resources, primarily forest lands, were observed as well as the type of land susceptible to primary erosion, including the level of urbanization and level of agricultural land. The obtained results clearly showed changes in forestation within the 43-year-long period, as well as changes in primary erosion and urbanization, while at the level of agricultural land, slight changes were found. The paper also involved transition of social factors from 1969 to 2012, expressed as a change between the earth and forest layer.

## 1. Introduction

The CORINE land cover is a European Environmental Agency project (Coordination of Information on the Environment, Land Cover, CLC), which started in 1985. The project includes a computerized and digitalized land cover of the 27 member states and other European states which are included in some phase of the project.

Standard CORINE records of land cover mapping are based on computer processing technology of satellite images with the use of ancillary data (topographic maps, aerial photo images, thematic maps). The records include analysis and identification of characteristics of objects on Earth, through colors, structures, textures, patterns, and relationships with other objects represented in the recordings. Satellite recordings and aerial photo imageries were used as the main source of mapping data. The recordings are mostly taken from the reference year +/− one year, with the minimum mapping unit of 25 ha. In the topographic maps 1 : 25,000, detectable size in meters is 25 meters. In the area of Prokuplje Municipality, the observations were done within the period of 43 years. With the help of time scales the connection between social and natural changes was established. The changes from 1969 to 2012 were observed through three processes: forestation, erosion, and urbanization.

To establish the correct prospect in observation of a particular territory, it is sufficient to know a single parameter. But if we observe more parameters, we can establish more conditioned relations. Modern technologies that observe geospatial data, with a GIS analysis involved, replace the traditional ones that observe a particular geospace (see [1]). In order to predict future conditions, we use the time scale, which presents a particular geographic territory monitoring (see [2]). During the period after World War II, this region faced two big transformations in social systems. The first was the change from capitalism to self-management socialism [3]. Social reforms were put into practice with the one from 1961 to 1969 as the most important [4], dealing with sudden industrialization on the territory of the former Socialist Federal Republic of Yugoslavia. The main problem was the fact that the entire population had already moved into factories and initiated urban migrations. The 1969 topographic maps can demonstrate the tendencies and conditions at the time and the initial screening of agricultural and social activities (see [5]). Until 1969, forests had been cut for obtaining low-category land. The land then was prepared for crops. Three basic factors were observed in monitoring of the forest land. A land structure was sampled from the 1 : 25,000 topographic maps; then the function of forestation expressed in percentage in relation to the entire municipality area was observed (see [6]). Three types of forests (broad leaved, coniferous, and the mixed type) were followed. The total forest area in 1969 was 21.4%. Contrary to forestation, the factors of human activity are represented by two other factors, urbanization and migrations from rural to urban settlements. Potential primary erosion is directly linked to agricultural production and population (see [7]). Without agricultural production there is no primary erosion, but it is the opposite process that is involved, called forestation [8]. Potential primary erosion is not real erosion, because the real one is related to a number of other factors.

## 2. Study Area and Demographic Characteristics of the Prokuplje Municipality

The Municipality of Prokuplje is located in the south eastern part of central Serbia in the Toplica region, in a valley, among hill sides and massive mountains of the middle part of the Toplica basin, between 43.00° and 43.4° of northern geographic latitude and 21.4° and 21.58° of eastern geographic longitude. It embraces an area of 758300000 m^2^ (see [9]). To the north, it borders the municipalities of Aleksinac and Krusevac, the municipalities of Blace and Kursumlija to the west and southwest, and the municipality of Bojnik to the south, while to the east it borders the municipalities of Zitoradja and Merosina. There are a motorway corridor and the Belgrade-Nis-Pristina railway, including six regional road tracks linking Prokuplje Municipality with nearby places. The distance from the city of Nis is 27 km, around 50 km from Leskovac and 250 km from the Serbian capital Belgrade. The river Toplica, 130 km long, flows through the central part of Toplica valley and through the Municipality of Prokuplje.

Thanks to the favorable geographic position and the terrain configuration, the most important Central Balkans Transversal partly runs through Toplica, over Kosovo and Metohija, creating the shortest route between the Vardar-Morava Valley and the Adriatic Sea. The route has historic, economic and cultural importance. The largest settlements, including Prokuplje, were developed in the Toplica valley as well as in the valley of the Toplica River tributaries, while mountains Radan and Veliki Jastrebac surround the area of the municipality to the north (see [10]).

There are 48,501 inhabitants living in the 107 settlements of Prokuplje Municipality. Some 57% of the inhabitants live in the town. The Municipality has 104 cadaster municipalities, 97 place communities with the average population rate of 466 per settlement (resource: Population Census, 2002). The largest population is in Prokuplje (27,673), while the smallest is in the settlement of Jovine Livade, with a population of 11 inhabitants. In the interval between the two censuses some settlements were vacant, with 0 inhabitants.

## 3. Material and Methods

**Figure 1:**
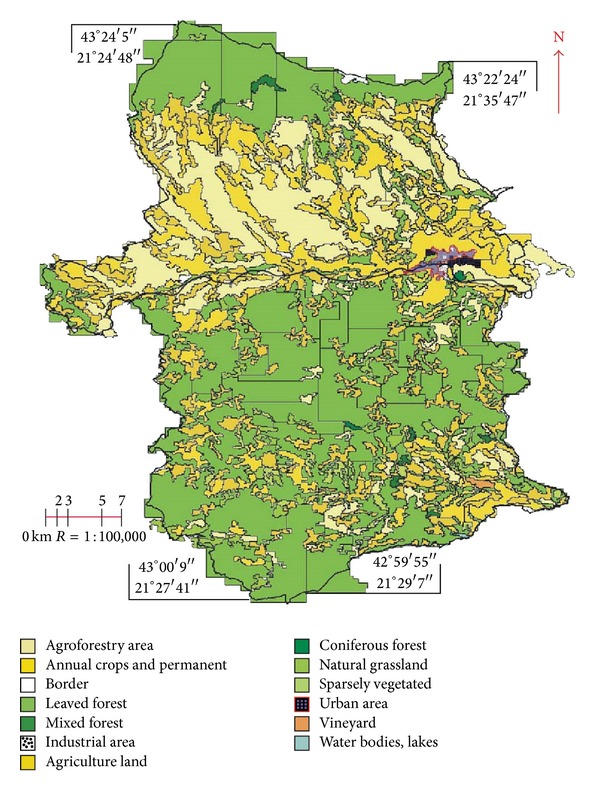
Digitized map of land cover on the territory of the Prokuplje Municipality in 1969.

In the analysis of sections of Prokuplje Municipality, the data obtained from analogous topographic maps were used. The topographic maps were in 1 : 25,000 scale, for the following sections: section 582 Nis: 1-4, 3-2, 1-3, 3-1, 3-3, and 3-4, 581 Kursumlija: 4-1, 2-4, 4-2, and 4-4. The topographic maps were scanned, digitized, and prepared in digital form (see [11]). By digitization, the analogue methods were substituted with the digital ones by using the GIS software Geo Media Professional 6.1 and Global Mapper 14. Then the digital data were transformed into vectors. The former raster maps became the vector-based maps (see Figure 1, the vector-based map in 1969).

**Figure 2:**
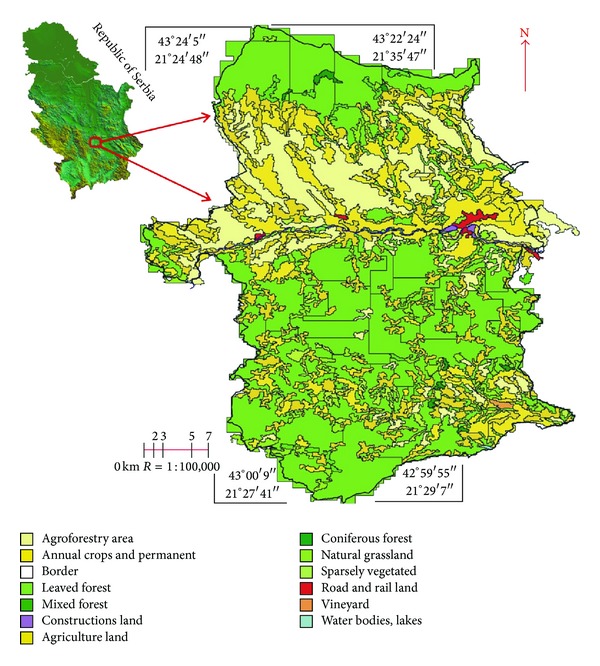
Digitized map of land cover on the territory of the Prokuplje Municipality in 2012.

Then, CORINE satellite recordings were downloaded from the official CORINE Project site (Figure 2). Land cover maps were obtained by using CORINE-like approach (see [12]). The primary erosion was calculated as presented in [12].

### 3.1. CORINE-Like Approach and Subpixel Data Analysis

Standard CORINE land cover map is based on computer supported satellite recordings, visual interpretation with the use of topographic map, aerial images, theme map, and field campaign (see [13]). The CORINE-like approach is based on the analysis of object feature recognition, including colors, structure, texture, patterns, and other object connections where objects of interest are in the recording (see [14]). The result of mapping implies a continuous set of vector data with the topology of a polygon. The data can be displayed in the raster format. As the basic source of mapping, Landsat/ETM recordings in the national coordinate system were used. The CORINE land cover maps were downloaded until 2012, with a tolerance of one-year gap. The minimum map unit is 25 ha and the minimum linear map width is 100 m (addendum of CORINE project). The proportion makes way for comparatively easy updating on a regular basis. The land cover map unit is equal to the area of homogenous earth layer consisting of a set of smaller homogenous areas which represent some more complex earth layer structure (see [15]). The land cover map unit should always be easily recognized in relation to the surrounding units and time stable enough. The linear unit of land cover map width is 100 m and it fits into the proportion of 1 mm of map proportion. The CORINE nomenclature has been explained in the CORINE Land Cover Technical Guide 1993 (see [16]). It is the physical and the physiognomic nomenclature of the Earth layer relevant to environmental monitoring. The earth layer categories are differentiated and categorized in the hierarchy of three levels. The first layer classes are artificial, agricultural, seminatural areas, forests, wet soil, and water areas. The second layer involves 15 and the third, most detailed one, includes 44 different earth layers. Every class has been represented by a three-digit code, each digit representing the proper leveled class place in the corresponding hierarchy. CORINE terminology is deeply connected with the process of visual recording interpretation, mapping proportion, and the minimum mapping unit.

The basic method in evaluating the quality and quantity of the land in Prokuplje Municipality was the analysis of subpixel data from the topographic maps and CORINE Land Cover Map. The method of subpixel mapping detail (described in [17, 18]) is a solution for subpixel data location with sufficient accuracy in the recording. This method is already applied in some satellite recordings. Mapping the borderline with subpixel accuracy can be achieved with subpixel mapping, where the finer resolution data are extracted from the original image, while at the same time the spatial location of the biophysical phenomena within the original pixels is obtained. This method can be applied on the digital maps of the observed territory as well. Such a method is called subpixel analysis in the pixel-swapping method. In the satellite image, this method is called soft classification, and it is a representation of the appearance in each pixel individually. The next step is to determine the location of subpixel component in each pixel and then pixel-swapping algorithm was applied. The spatial resolution of the original pixel is divided into 16 (4 × 4) subpixels. The algorithm is based on the geostatistical method for classifying subpixels. For every subpixel the parameter of affiliation *A*_*i*_ was calculated. This variable represents the weighting function of the event representation percent [19, 20]:

(1) $$\begin{array}{c} A_{i}=\overset{n}{\underset{j=1}{\sum }}\lambda_{ij}z\left(u_{j}\right), \end{array}$$

where *n* is the number of neighbor pixel, *z*(*u*_*j*_) is event representation percent in *j* pixel, and *λ*_*ij*_ is the weight of the pixel which depends on the distance within the pixel, and

(2) $$\begin{array}{c} \lambda_{ij}=\exp\left(\frac{-h_{ij}}{a}\right), \end{array}$$

where *h*_*ij*_ is a distance between the pixels, (*i*) for which the parameter of affiliation is calculated, neighbor pixels on the location (*j*), and (*a*) is nonlinear parameter of *t* exponential method. Similar soft combined subpixel method is applied in the topographic maps that are digitized; events within the pixels are given in the division (4 × 4) subpixels, 32 (6 × 6), 64 (8 × 8), and 256 (16 × 16). This is done to achieve better accuracy because the quality of the digitized map sheets is weaker than land cover maps obtained by using CORINE-like approach.

### 3.2. Estimation of the Trend of Meteorological Variables on the Territory of the Prokuplje Municipality

In addition to anthropogenic factors that have impact on certain changes in the land and the forest belt, it is necessary to examine the influence of meteorological variables on a given territory and to compare the obtained data with other changes that have occurred. We gathered data from the meteorological station in Prokuplje (http://www.hidmet.gov.rs/), and we gave an estimation of the climatic changes based on two parameters: temperature (max, min, average) and average amount of rainfall (the parameters measured on the meteorological station in Prokuplje are temperature, rainfall and pressure). The period of observation is from 1974 to 2012 (39 time series, the first recorded data of meteorological parameters for the Prokuplje municipality were from 1974). For the estimation of the trend, Mann-Kendall trend test was used (see [21, 22]), with significance level alpha = 0.05. The used softwer is XL-stat (http://www.xlstat.com/en/). The test data are given in Table 1.

**Figure 3:**
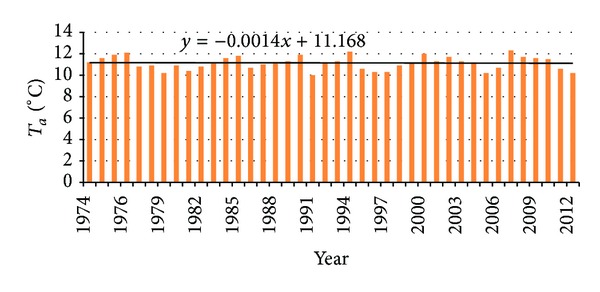
Linear tendency (trend) equation for the period from 1974 to 2012 for average temperatures in meteorological station Prokuplje.

**Figure 4:**
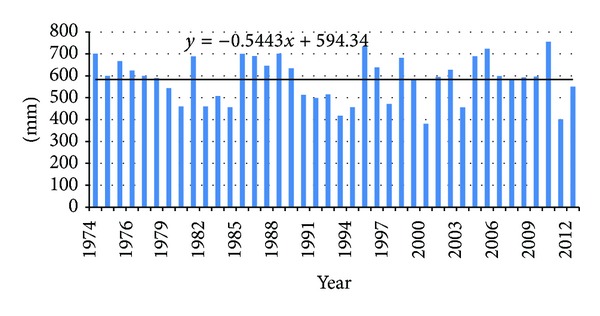
Linear tendency (trend) equation for period from 1974 to 2012 for average rainfall in meteorological station Prokuplje.

By analyzing Figure 3, it can be concluded that no trend is found for the minimum, maximum, and average temperature (H0 hypotesis is accepted or the trend is probably rejected). By analyzing Figure 4, where average rainfall data are given, the trend was not found either. Based on the obtained data, it is concluded that meteorological variables (measured in period 1974–2012) had no effect on climatic changes and distribution of forest and land cover on the territory of the municipality.

*Test Interpretation (Figure 3)*. Kendall's tau is 0.010.* P* value (two-tailed) is 0.942. Alpha is 0.05. H0: there is no trend in the series. Ha: there is a trend in the series. As the computed* P* value is greater than the significance level alpha = 0.05, one cannot reject the null hypothesis H0. For average temperature the risk to reject the null hypothesis H0 while it is true is 94.20%. The test interpretation of Mann Kendall trend test shows that there is no trend in temperature changes.

*Test Interpretation (Figure 4)*. Kendall's tau is −0.038.* P* value (two-tailed) is 0.744. Alpha is 0.05. H0: there is no trend in the series. Ha: there is a trend in the series. As the computed* P* value is greater than the significance level alpha = 0.05, one cannot reject the null hypothesis H0. For average of rainfall the risk to reject the null hypothesis H0 while it is true is 74.39%. The test interpretation of Mann Kendall trend test shows that there is no trend in rainfall changes.

## 4. Results and Discussion 

The changes of land cover uses are the result of rural to urban migrations, reclassification of rural areas to urban areas, deforestation, and use of ecologically incompatible technologies. During the past few decades, the study area borne witness to substantial decrease in population, stagnation of economic growth, and industrialization which had a negative impact on the region.

**Figure 5:**
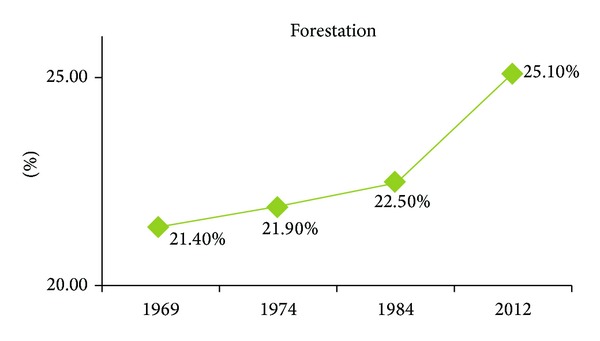
Forestation in the period from 1969 to 2012 for three types of forest: deciduous, conifirous, and mixed, derived from topographic maps (TK 1 : 25000, Section 582 NIs: 1-4, 3-2, 1-3, 3-1, 3-3, and 3-4, 581 Kursumlija: 4-1, 2-4, 4-2, and 4-4) and from Land Cover Map obtained by using CORINE-like approach in 2012.

**Figure 6:**
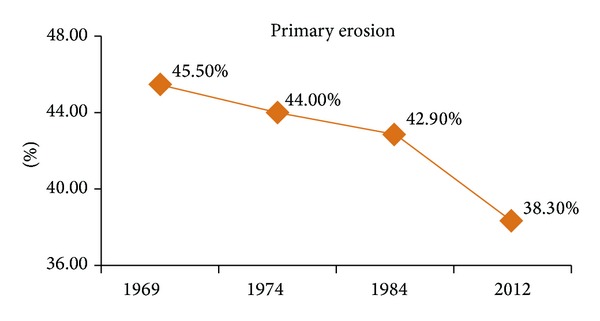
Primary erosion in the period from 1969 to 2012 for two types of land: agroforestry and the land mainly occupied with agriculture, derived from topographic maps (TK 1 : 25,000, section (582 Nis: 1-4, 3-2, 1-3, 3-1, 3-3, and 3-4, 581 Kursumlija: 4-1, 2-4, 4-2, 4-4) and from Land Cover Map obtained by using CORINE-like approach in 2012.

**Figure 7:**
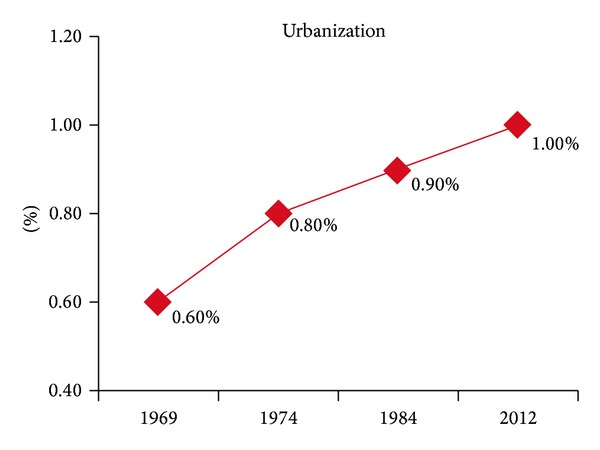
Urbanization in the period from 1969 to 2012 for urban area of the Prokuplje Municipality: derived from topographic maps (TK 1 : 25,000, section 582 Nis: 1-4, 3-2, 1-3, 3-1, 3-3, and 3-4, 581 Kursumlija: 4-1, 2-4, 4-2, and 4-4) and also from Land Cover Map obtained by using CORINE-like approach in 2012.

The study area covers 758300000 m^2^ and the changes were estimated from 1969 to 2012. Tables 2, 3, 4, and 5 and Figures 5, 6, and 7 show the statistical results of changes. The analogous 1 : 25,000 topographic maps used in research were first scanned and then the raster material was digitized in GeoMedia 6.1 Professional and Global Mapper software.

In 1969, the forestation was 21.40% (Figure 5), primary erosion was 45.50% (Figure 6, compared to the entire territory 0.60%), and urbanization was 0.6% (Figure 7).

Before 1969, the forestation must have been even lower, around 19% in 1953 (Census of Yugoslavia). The next analogous topographic map was made in 1974. Those data (Figures 5, 6, and 7) reveal 21.90%, forestation, 44.00%, potential primary erosion, and 0.80%, urbanization thus indicating that the five-year-long period was marked by increased forestation and urbanization which indicated rural migrations. The topographic map done in 1984, issued by the Military Geographic Institute, was the last analogous map used in the monitoring. The data in 1984 show the monitoring in a ten-year period, which is long enough to observe particular phenomena, especially the social changes as urbanization (see [23]). The data in 1984 are presented in Figures 5, 6, and 7: forestation was 22.50%, potential primary erosion was 42.90%, and urbanization was 0.90%.

Land Cover Maps obtained by using CORINE-like approach have a better data usability and easier analytic conversion within GIS. A raster form was downloaded from the official CORINE site, inserted into a shape file within the scope of Prokuplje Municipality. Digital precision with raster formats had the value of 0.5–1%. The precision of a CORINE land cover maps is 0.1% [24]. A Land Cover Map obtained by CORINE-like approach was the last used in monitoring of the Prokuplje Municipality and in 2012, the data were the following: forestation 25.10%, potential primary erosion 38.30%, and urbanization 1.0%, (Figures 5, 6, and 7).

In 1969, agricultural land (annual crops associated with permanent crops, land mainly occupied with agriculture and vineyards) on the territory of the Municipality had a great proportion in the total area (45.2%, Table 2). In 1974 there was a slight decrease (45.1%, Table 3), while in 1984 a slight increase continued (45.6%, Table 4). In the last observed year, 2012, on the territory of the Municipality the percentage of agricultural land was 45.9% (Table 5). Thus, it can be concluded that we have a slight changes in the percentage of agricultural land and evidently decreased vineyards (in 1969 vineyards were planted on 0.6% of land cover, in 1974, 1984, and 2012 they occupied 0.5%, 0.7%, and 0.3%, resp.).

Based on the obtained results during the 43-year-long period it can be concluded that the forestation increased greatly, which indicates the decreased number of settlements (according to the Census of the Republic of Serbia in 2013, numerous villages were abandoned) due to social changes in the Toplica region during the period from 1984 to 2012, [25].

Monitoring the Municipality of Prokuplje has shown the tendency of increased urbanization solely related to the central parts of the town, which indicates the constant rural to urban migrations. Namely, due to social changes in the Toplica region during the period from 1984 to 2012 the level of industrialization considerably decreased. Therefore, a constant migration of the population from this area is noticed, especially to Belgrade, the capital of Serbia (according to the Census of the Republic of Serbia in 2013, the depopulation on this territory amounted 10.1%).

Based on the presented data, it is surprising that at the time of sudden anthropogenic changes the number of settlements in the Toplica region decreases while the forest area increases, which is contrary to the processes in other developing countries (see [26]), since the increased urbanization largely affects rapid reduction of forested areas.

## 5. Conclusion 

According to the results obtained by monitoring, great changes can be observed in the total amount of forestation (Figure 5). The increase in total amount of forestation is clearly explained by the fact that forests have been returning to their initial habitats before 1969. The major change has occurred with the mixed type forests. These forests usually appear in agroforest land, indicating slow invasion in the territory of the formerly cultivated area; this happened within the 40-year-long time span. The data which show changes (forestation, urbanization, the primary erosion) in the 43-year-monitoring period point to three aspects of the observed territory of the Prokuplje Municipality. The conclusion is that the forests are slowly returning to their former habitats, because in 1931, there were about 51% of forests (the data from census of the Kingdom of Serbian Croatian and Slovenian). However, the changes in forestation were not due to climatic changes. Namely, we found no trend in climatic changes by observing two parameters: temperature and rainfalls (Figures 3 and 4). The data on urbanization were not available in the former census records, but it is presumed that in 1931, its level was about 0.5%. Urbanization is a process that is completely slowed down. There are 12,345 inhabitants in the Municipality of Prokuplje. By the Census in 1931, 79% of the population was rural. Due to the beginning of deforestation process, primary erosion began between the two world wars. The primary erosion stopped with agricultural activity of the population in this rural municipality. Now the population is in the process of migration towards larger centers. The agricultural activity shows small changes; it is mostly stagnant. Further depopulation, migrations, and unemployment may turn this region into an animal and plant oasis, capable of representing a tourist econational park.

## Figures and Tables

**Table 1:** Trends of four time series data from meteorological station Prokuplje with Kendall *τ* rank correlation. Significance level alpha is 0.05 or 5% of inputted data.

Station Prokuplje	Altitude (m)	Latitude (°)	Longitude (°)	Minimum maximum average *T* in °C 1974–2012, rainfall in mm	Kendall's τ	St. deviation *σ*
	274 m	43.24	21.59	5.56	0.010	1.057
				17.63	0.321	0.737
				11.13	0.044	0.618
				755.400 mm	−0.038	100.322

**Table 2:** The percentage and area of types of land on the territory of Prokuplje Municipality in m^2^ in 1969.

Agroforestry areas	19%	144074000 m^2^
Annual crops associated with permanent crops	18.1%	137254000 m^2^
Broad leaved forest	19.5%	147869000 m^2^
Coniferous forest	1.6%	12133000 m^2^
Industrial area	0.3%	2275000 m^2^
Land principally occupied by agriculture	26.5%	200950000 m^2^
Mixed forest	0.3%	2275000 m^2^
Natural grassland	9.1%	69005000 m^2^
Sparsely vegetated area	4.5%	34124000 m^2^
Vineyards	0.6%	4550000 m^2^
Urban area	0.3%	2275000 m^2^
River Toplica	0.2%	1516000 m^2^

Total Sum	100%	758300000 m^2^

**Table 3:** The percentage and area of types of land on the territory of Prokuplje Municipality in m^2^ in 1974.

Agroforestry areas	18.1%	137254000 m^2^
Annual crops associated with permanent crops	18.7%	141802000 m^2^
Broad leaved forest	19.7%	149385000 m^2^
Coniferous forest	1.8%	13649000 m^2^
Industrial area	0.4%	3033000 m^2^
Land principally occupied by agriculture	25.9%	196400000 m^2^
Mixed forest	0.4%	3033000 m^2^
Natural grassland	9.3%	70522000 m^2^
Sparsely vegetated area	4.4%	33365000 m^2^
Vineyards	0.5%	3792000 m^2^
Urban area	0.4%	3033000 m^2^
River Toplica	0.2%	1516000 m^2^
Water Bodies, Lakes	0.2%	1516000 m^2^

Total Sum	100%	758300000 m^2^

**Table 4:** The percentage and area of types of land on the territory of Prokuplje Municipality in m^2^ in 1984.

Agroforestry areas	16.2%	122845000 m^2^
Annual crops associated with permanent crops	18.2%	138011000 m^2^
Broad leaved forest	20%	151660000 m^2^
Coniferous forest	1.7%	12891000 m^2^
Industrial area	0.4%	3033000 m^2^
Land principally occupied by agriculture	26.7%	202466000 m^2^
Mixed forest	0.8%	6066000 m^2^
Natural grassland	9.0%	68247000 m^2^
Sparsely vegetated area	5.2%	39432000 m^2^
Vineyards	0.7%	5308000 m^2^
Urban area	0.5%	3792000 m^2^
River Toplica	0.2%	1516000 m^2^
Water Bodies, Lakes	0.4%	3033000 m^2^

Total Sum	100%	758300000 m^2^

**Table 5:** The percentage and area of types of land on territory of Prokuplje Municipality in m^2^ in 2012.

Agroforestry areas	10.9%	82654000 m^2^
Annual crops associated with permanent crops	17.6%	133461000 m^2^
Broad leaved forest	21.5%	163035000 m^2^
Coniferous forest	1.5%	11375000 m^2^
Constructions sites land	0.3%	2275000 m^2^
Land principally occupied by agriculture	28%	212324000 m^2^
Mixed forest	2.1%	15924000 m^2^
Natural grassland	10.4%	78863000 m^2^
Sparsely vegetated area	5.2%	39432000 m^2^
Vineyards	0.3%	2275000 m^2^
Road and rail networks with associated land	0.7%	5308000 m^2^
River Toplica	0.2%	1516000 m^2^
Water Bodies, Lakes	0.3%	2275000 m^2^
Urban areas	1%	7583000 m^2^

Total Sum	100%	758300000 m^2^

## Internet Sources

Internet source 1 was http://www.globalmapperforum.com/forums/forum.php/, retrieved 1 February 2014. Internet source 2 was http://www.intergraphforums.com/support/forum/2, retrieved 7 February 2014.

## Software

Software used was XL-stat, Global Mapper V14 xx, and GeoMedia Professional 6.1.
